# Stability of silver nanoparticle monolayers determined by in situ streaming potential measurements

**DOI:** 10.1007/s11051-013-2076-5

**Published:** 2013-10-31

**Authors:** Maria Morga, Zbigniew Adamczyk, Magdalena Oćwieja

**Affiliations:** Jerzy Haber Institute of Catalysis and Surface Chemistry, Polish Academy of Sciences, Niezapominajek 8, 30-239 Kraków, Poland

**Keywords:** Deposition of silver particles, Release kinetics of silver nanoparticles, Monolayers of silver particles, Streaming potential of silver particle monolayers

## Abstract

**Abstract:**

A silver particle suspension obtained by a chemical reduction was used in this work. Monolayers of these particles (average size 28 nm) on mica modified by poly(allylamine hydrochloride) were produced under diffusion-controlled transport. Monolayer coverages, quantitatively determined by atomic force microscopy (AFM) and SEM, was regulated by adjusting the nanoparticle deposition time and the suspension concentration. The zeta potential of the monolayers was determined by streaming potential measurements carried out under in situ (wet) conditions. These measurements performed for various ionic strengths and pH were interpreted in terms of the three-dimensional (3D) electrokinetic model. The stability of silver monolayers was also investigated using streaming potential and the AFM methods. The decrease in the surface coverage of particles as a function of time and ionic strength varied between 10^−1^ and 10^−4 ^M was investigated. This allowed one to determine the equilibrium adsorption constant *K*_a_ and the binding energy of silver particles (energy minima depth). Energy minima depth were calculated that varied between −18 kT for *I* = 10^−1 ^M and −19 kT for *I* = 10^−4^ for pH 5.5 and *T* = 298 K. Our investigations suggest that the interactions between surface and nanoparticles are controlled by the electrostatic interactions among ion pairs. It was also shown that the in situ electrokinetic measurements are in accordance with those obtained by more tedious *ex situ* AFM measurements. This confirmed the utility of the streaming potential method for direct kinetic studies of nanoparticle deposition/release processes.

**Graphical Abstract:**

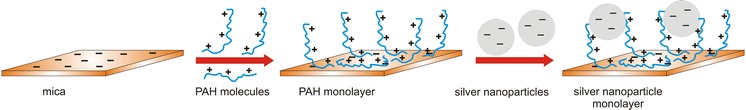

## Introduction

Silver nanoparticles and their monolayers on solid substrates have found a wide range of practical applications for producing photonic and antireflective materials (Nishioka et al. 2009), in catalysis (Pradhan et al. 2002), as analytical sensors in SERS spectroscopy (Chen et al. 2007; Kaczor et al. 2010), metal-enhanced fluorescence (MEF) (Aslan et al. 2005), in immunosensing biologic probes and markers (Cai et al. 2002; Liu et al. 2006). Due to the excellent biostatic properties, they are also used to modify surfaces of various materials, in particular fibers or polymers (Flores et al. 2010; Kong and Jang 2008; Tang et al. 2012), applied in manifold consumer products such as clothes, laboratory and surgical gowns, dressing bandages (Kulthong et al. 2010), etc.

In order to control the preparation of silver monolayers for such wide range of applications, a thorough knowledge of particle interactions with various surfaces and deposition/release mechanisms is required.

Particle deposition processes are often studied using indirect experimental methods such as ellipsometry and reflectometry (Reiter et al. 1992), total internal reflection fluorescence (Bharill et al. 2011), UV–Vis surface plasmon adsorption (Bar et al. 1996), atomic force microscopy (AFM) (Yang et al. 2007), scanning electron microscopy (SEM), or quartz crystal microgravimetry (QCM) (Bandyopadhyay et al. 1997). However, a disadvantage of these methods is that they become only sensitive for higher coverage, preferably close to the saturation coverage, and often work under *ex situ*, dry conditions.

Therefore, the aim of this work is a thorough electrokinetic study of silver nanoparticle monolayers on PAH-covered mica and the investigation of the release kinetics of particles from such monolayers. This is determined using the streaming potential technique, which enables one to conduct experiments under in situ conditions. In our studies, monolayers of controlled coverage and homogeneity are produced in the diffusion controlled self-assembly process by adjusting the suspension concentration, pH, ionic strength, and the deposition time (Adamczyk et al. 2011b; Dąbkowska and Adamczyk 2012; Morga et al. 2012; Oćwieja et al. 2011, 2012b, 2013). These measurements are interpreted in terms of the 3D electrokinetic model based on exact solutions of the Stokes and Poisson–Boltzmann equations governing the flow and electrostatic potential fields (Adamczyk et al. 2010, 2011b). This model was previously applied for monodisperse latex particles (Zaucha et al. 2011), polyelectrolytes (Morga and Adamczyk 2013), proteins (Dąbkowska and Adamczyk 2012; Wasilewska and Adamczyk 2011), and iron oxide particles (Adamczyk et al. 2011b; Dąbkowska and Adamczyk 2012; Morga et al. 2012). In this way, one can quantitatively determine silver particle desorption kinetics over extensive time periods that allow to calculate the biding energy for various physicochemical parameters such as ionic strength a pH. This is a unique possibility not accessible to others, indirect experimental methods.

It should be mentioned that up to our knowledge, no such electrokinetic characteristics of silver nanoparticle monolayers on solid surfaces were reported in the literature.

## Experimental

### Materials

All chemical reagents used in the experiments (silver nitrate, trisodium citrate, sodium chloride, sodium hydroxide, and hydrochloric acid) were commercial products of Sigma Aldrich and applied directly without further purification.

Natural ruby mica sheets were purchased from Continental Trade and used as a solid substrate for the colloidal particle adsorption. The solid pieces of mica were freshly cleaved into thin fragments of desired area and used in each experiment without any pretreatment. Ultrapure water obtained using the Milli-Q Elix&Simplicity 185 purification system from Millipore SA Molsheim, France was used to prepare all solutions and synthesize colloidal suspensions.

The cationic polyelectrolyte, poly(allylamine hydrochloride) (PAH), having a molecular weight of 70 kDa was purchased from Polysciences and used as supporting layer for silver nanoparticle deposition.

Silver nanoparticle suspensions were synthesized by a chemical reduction of AgNO_3_ using trisodium citrate, which has been widely applied for the preparation of various metallic nanoparticles (Kamyshny and Magdassi 2009) and described in details in our previous work (Oćwieja et al. 2013). Briefly, a sample of silver nitrate (200 mg) was dissolved in distilled water to obtain silver ions concentration of 1.18 mM and then heated to 88 °C under stirring (the rate of stirring 300 rpm). Afterward, 4 mL of 1 % trisodium citrate solution was added rapidly to the silver solution. The mixture was kept at 88 °C for 35 min with continuous stirring. After this period of time, the silver suspension was immediately cooled to the room temperature. The silver sol was purified from ionic excess using a stirred membrane filtration cell (Millipore, model 8400) with a regenerated cellulose membrane (Millipore, NMWL 100 kDa). The procedure was continued until the conductivity of the supernatant solution stabilized at 15–20 μS/cm.

### Methods of silver nanoparticle suspension and monolayer characterization

The weight concentration of the particle suspension was determined using a high precision densitometer: DMA 5000M (Anton Paar).

UV–Vis extinction spectrum was measured using the Shimadzu UV-1800 spectrometer.

Diffusion coefficients of silver nanoparticles (hydrodynamic diameter) were determined by dynamic light scattering (DLS) using the Zetasizer Nano ZS from Malver company.

The microelectrophoretic mobility of the nanoparticles was measured by the laser Doppler velocimetry technique (LDV) using the same equipment and additionally using the Brookhaven Zeta Pals apparatus.

The morphology of silver nanoparticles was investigated using the JEOL JSM-7500F microscope working in transmission mode. Samples for this examination were prepared by dispersing a drop of the silver colloid on a copper grid which was covered by a carbon film. Furthermore, the scanning electron microscope JEOL JSM-7500F was used to determine the coverage of silver monolayers. To ensure a sufficient conductivity, before the measurement, the silver nanoparticles samples were covered with a thin layer of chromium.

Independently, the surface concentration of silver particles on the modified mica substrate was quantitatively determined using AFM. These measurements were carried out using the NT-MDT Solver Pro instrument with the SMENA SFC050L scanning head. Imaging was done in the semicontact mode using silicon probe (polysilicon cantilevers with resonance 120 kHz ± 10 %, typical curvature radius tip was 10 nm, cont angle was <20°).

The zeta potentials of PAH-covered mica and silver particle monolayers were determined via streaming potential measurements using a home-made cell previously described (Adamczyk et al. 2010; Zembala et al. 2001). The main part of the cell is a parallel plate channel of dimensions 2*b*_*c*_× 2 *c*_*c*_ × *L* = 0.027 × 0.29 × 6.2 cm, formed by mica sheets separated by a perfluoroethylene spacer. A laminar flow of the electrolyte was forced by a regulated hydrostatic pressure difference. The streaming potential *E*_s_ was measured using a pair of Ag/AgCl electrodes as a function of the hydrostatic pressure difference Δ*P* which was driving the electrolyte flow through the channel. The cell electric conductivity *K*_e_ was determined by Pt electrodes. Knowing the slope of the *E*_s_ versus Δ*P* dependence, the apparent zeta potential of substrate surface (*ζ*_*i*_) can be calculated from the Smoluchowski relationship:

1 $$\zeta_{i} = \frac{\eta L}{{4\varepsilon b_{c} c_{c} R_{\text{e}} }}\left( {\frac{{\Updelta E_{\text{s}} }}{\Updelta P}} \right) = \frac{{\eta K_{\text{e}} }}{\varepsilon }\left( {\frac{{\Updelta E_{\text{s}} }}{\Updelta P}} \right),$$

where *η* is the dynamic viscosity of the solution; *ε* is the dielectric permittivity; *R*_e_ is the electric resistance of the cell governed mainly by the specific conductivity of the electrolyte in the cell, and *K*_e_ is the specific conductivity of the cell, connected with the electric resistance via constitutive relationship:

2 $$R_{e} = \frac{L}{{\Updelta S_{\text{c}} K_{\text{e}} }} = \frac{L}{{\Updelta S_{\text{c}} (K'_{\text{e}} + K_{\text{s}} )}},$$

where Δ*S*_*c*_ = 2*b*_*c*_ × 2 *c*_*c*_ is the channel cross-sectional area; *K′*_e_ is the specific conductivity due to electrolyte, and *K*_s_ is the surface conductivity, depending in general on the channel shape. It is worth mentioning that the correction concerning the surface conductivity was practically negligible for the ionic strength of electrolytes above 10^−3^ M.

The procedure of preparing silver nanoparticle monolayers on PAH-covered mica was as follows:

i. Reference measurement of the streaming potential for bare mica in pure electrolyte of various ionic strengths a pH was done.
ii. A supporting PAH monolayer of controlled coverage was formed in situ under diffusion-controlled transport. This was realized by filling the electrokinetic cell with the PAH solution of an appropriate concentration (0.2–5 mg L^−1^), for an appropriate time period (1–15 min).
iii. The cell was flushed with pure electrolyte of the same ionic strength and pH, and the streaming potential for PAH covered mica is measured.
iv. Silver particle monolayers of controlled coverage were deposited in situ under diffusion-controlled conditions by filling the electrokinetic cell with the suspension of appropriate concentration (20–250 mg L^−1^) for an appropriate time period (1–60 min).

## Results and discussion

### Silver particle and PAH characteristics

The weight concentration of the stock silver suspension was determined by the densitometer as described in our previous works (Oćwieja et al. 2011, 2012b). The supernatant solution used in these measurements was acquired by membrane filtration. The stock silver particles suspension having the concentration 550 mg L^−1^ was diluted prior to experiments.

The size distribution and morphology of the silver particles were determined from AFM images and SEM micrographs. The nanoparticle diameter (*d*_p_) was calculated as the average value from two perpendicular directions and from the surface area of particles, as described before (Oćwieja et al. 2011, 2013). According to the histogram obtained from SEM micrographs, the mean diameter of particles was 28 nm with a standard deviation of 4 nm.

The average size distribution of particles was also determined by AFM. Silver particles were deposited from a dilute suspension (20 mg L^−1^), pH = 5.5, *I* = 10^−2 ^M for 15 min, on a mica sheet precovered by a supporting PAH layer. The size of the particles was determined using the Nova 1152 software which is directly coupled to the AFM microscope. The average size of particles determined from the histogram was 29 nm, with a standard deviation of 5 nm.

The average particle size (hydrodynamic diameter, *d*_H_) was also determined via diffusion coefficient (*D*) measurements performed using the DLS method. Knowing the diffusion coefficients, one can determine the hydrodynamic diameter using the Stokes–Einstein relationship:

3 $$d_{\text{H}} = \frac{kT}{3\pi \eta D},$$

where *k* is the Boltzman constant; *T* is the absolute temperature, and *η* is the dynamic viscosity of the solvent. The hydrodynamic diameter can be interpreted as the size of an equivalent sphere having the same hydrodynamic resistance coefficient as the particle. The advantage of using this quantity in comparison to the diffusion coefficient is that it is independent of temperature and liquid viscosity, so it is an appropriate parameter for analyzing a suspension stability under various conditions (Oćwieja et al. 2013).

For the sake of convenience, PAH and silver particle sizes, determined by various technique, are collected in Table 1.

**Table 1:** Bulk physicochemical characteristics of PAH molecules and silver nanoparticles

Physicochemical properties	Value	Remarks
PAH		
Specific density (g cm^−3^)	1.15	Adamczyk et al. (2006)
Diffusion coefficient (cm^2^ s^−1^)	1.51 × 10^−7^	Determined by DLS for *T* = 298 K, pH 5.5 *I* = 10^−2 ^M (Morga and Adamczyk 2013)
Hydrodynamic diameter (nm)	32.5 ± 4	Calculated from Eq. (3). For *T* = 298 K, pH 5.5 *I* = 10^−2^ M (Morga and Adamczyk 2013)
Geometrical cross-section area (nm^2^)	155	Adamczyk et al. (2006)
Silver		
Specific density (g cm^−3^)	10.49	Literature data (Fuertes et al. 2009)
Average particle size (nm)	28 ± 4	From size distribution derived from TEM micrographs (Oćwieja et al. 2013)
Average particle size (nm)	29 ± 5	From size distribution derived from AFM images (Oćwieja et al. 2013)
Diffusion coefficient (cm^2^ s^−1^)	1.48 × 10^−7^	Determined by DLS for *T* = 298 K, pH 6.2 *I* = 5 × 10^−2^–0.03 M NaCl
Hydrodynamic diameter (nm)	29 ± 5	Calculated from Eq. (3) (Oćwieja et al. 2013)
Plasmon absorption maximum (nm)	400	Measured for pH 6.2 *I* = 10^−2^ M NaCl and silver sol concentration, *c*_*b*_ = 5–25 mg L^−1^ (Oćwieja et al. 2013)
Geometrical cross-section area (nm^2^)	615	Calculated from geometry (Oćwieja et al. 2013)

The electrophoretic mobility *μ*_e_ of PAH molecules and silver nanoparticles, defined as the average translation velocity under given electric field, was determined by micro-electrophoresis. This quantity is of an essential significance for characterizing particle stability and interactions with surfaces governing deposition/release processes.

Knowing the electrophoretic mobility and the hydrodynamic diameter, one can determine the effective (uncompensated) charge per polyelectrolyte molecule or colloidal particle *q* from the Lorentz–Stokes relationship:

4 $$q = \frac{kT}{D}\mu_{\text{e}} = 3\pi \eta d_{\text{H}} \mu_{e} ,$$

where *μ*_e_ is the averaged migration velocity of PAH molecules in the uniform electric field *E*.

Equation (4) can be directly used for calculations of the average number of elementary charges per molecule considering that *e* = 1.602 × 10^−19 ^C, thus:

5 $$N_{\text{c}} = \frac{30\pi \eta }{1.602}d_{H} \mu_{e} ,$$

where *N*_c_ is expressed as the number of elementary charge *e* per molecule; *η* is the solution dynamic viscosity expressed in g (cm s)^−1^; *d*_H_ is hydrodynamic diameter of a particle or molecule, expressed in nm, and *μ*_*e*_ is electrophoretic mobility expressed in μm cm (V s)^−1^.

The number of uncompensated (electrokinetic) charges calculated in this way for PAH molecules and silver nanoparticles for ionic strength ranging from 10^−4^ to 0.15 M NaCl, and various pH are collected in Table 2.

**Table 2:** The electrophoretic mobility, the number of elementary charges, and the zeta potentials of PAH molecules and silver nanoparticles for various pH and ionic strengths

Ionic strength (M)	pH	*d*_H_ (nm)	*κd*_H_	*μ*_*e*_ (μm cm (V s)^−1^)	*N*_c_	*ζ* (mV) Henry’s model	*ζ* (mV) Smoluchowski’s model
PAH							
0.0001	4.0	34	0.51	5.1	84	95	64
	5.5	35	0.54	4.7	79	89	60
	9.0	38	0.62	2.4	46	44	30
0.001	3.5	33	1.26	4.5	72	77	54
	4.0	33	1.26	4.5	72	77	54
	5.5	33	1.68	4.4	67	79	56
	9.0	35	1.79	2.4	39	42	30
0.01	3.5	32	4.16	4.2	66	72	54
	4.0	32	4.16	4.2	66	72	54
	5.5	32	5.32	4.1	63	66	51
	9.0	37	6.01	2.1	36	34	27
0.15	3.5	31	15.5	2.7	41	39	34
	4.0	31	15.5	2.7	41	39	34
	5.5	32	19.9	2.8	42	40	36
	9.0	35	21.9	1.2	19	16	15
Silver							
0.0001	5.5	29	0.46	−3.8	−65	−79	−53
	6.2	29	0.46	−3.9	−64	−81	−54
	9.0	29	0.46	−4.1	−68	−86	−58
0.001	3.5	29	1.45	−2.6	−43	−52	−37
	5.5	29	1.45	−3.2	−53	−64	−45
	6.2	29	1.45	−3.3	−54	−80	−46
	9.0	29	1.45	−3.5	−58	−71	−49
0.01	3.5	29	4.59	−2.4	−40	−44	−33
	5.5	29	4.59	−2.8	−46	−51	−39
	6.2	29	4.59	−2.8	−47	−53	−40
	9.0	29	4.59	−3.1	−50	−57	−43

### Silver monolayers on mica—streaming potential studies

As mentioned, the streaming potential method enables in situ measurements, which was exploited in our work to monitor the formation of the PAH-supporting layer and the silver nanoparticle monolayer under various physicochemical conditions.

In the first stage of the experimental procedure, described above, the PAH monolayer of a controlled coverage was produced directly in the streaming potential cell under diffusion-controlled conditions. The nominal coverage of PAH (*θ*_PAH_) was calculated considering the diffusion-controlled transport from finite volumes where the adsorption kinetics is governed by the equation (Adamczyk et al. 2006):

6 $$\theta_{\text{PAH}} = S_{\text{g}} h\left[ {1 - \frac{8}{{\pi^{2} }}\sum\limits_{i = 1}^{\infty } {\frac{{e^{{ - \frac{{\left( {2i - 1} \right)^{2} \pi^{2} D_{\text{p}} }}{{4h^{2} }}t}} }}{{\left( {2i - 1} \right)^{2} }}} } \right]n_{\text{p}} ,$$

where *S*_g_ is the characteristic cross-section of the PAH molecules determined to be 155 nm^2^ (Adamczyk et al. 2006); 2*h* is the distance between the two mica sheets forming the channel; *D*_p_ is a diffusion coefficient of PAH; *t* is adsorption time; *n*_p_ is the polyelectrolyte number concentration, expressed in molecules per cm^3^, connected with the its concentration expressed in mg L^−1^ by the following relationship: $n_{p} = 10^{ - 6} c_{b} \frac{{A_{\text{v}} }}{{M_{w} }}$ (Adamczyk et al. 2006), *A*_v_ = 6.023 × 10^23^ is the Avogadro number and *M*_w_ is the molar mass of PAH.

Afterward, the cell was flushed with pure electrolyte; the streaming potential of PAH-covered mica was measured, and the zeta potential was calculated using Eq. (1).

**Fig. 1:**
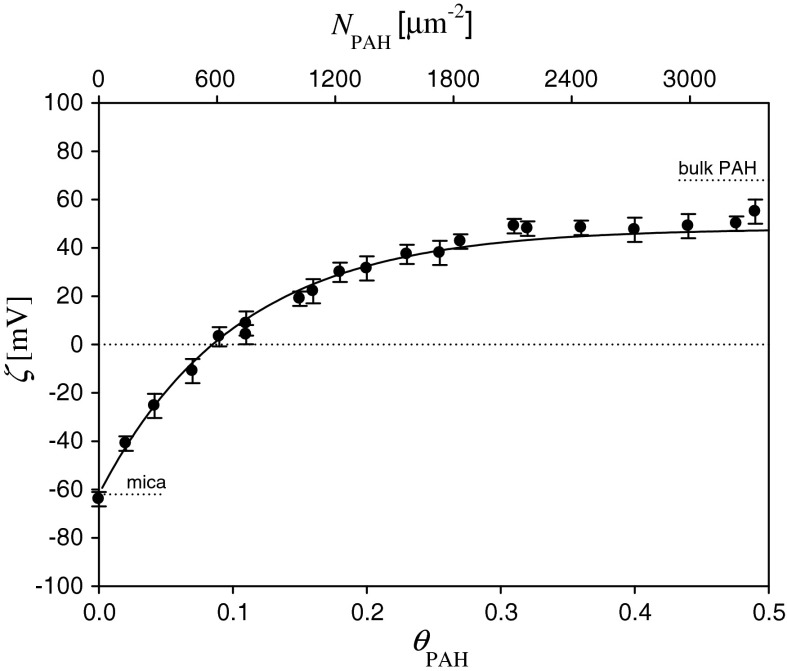
The dependence of the zeta potential of mica *ζ* on the nominal coverage of PAH, *θ*_PAH_. The *points* denote experimental results obtained from the streaming potential measurements. PAH adsorption was carried out for pH 5.5, *I* = 10^−2^ M. The *solid line* denotes the exact theoretic results calculated from the 3D electrokinetic model, Eqs. (7, 8) for *ζ*_PAH_ = 66 mV

The dependence of the zeta potential of mica on the coverage of PAH derived from these measurements for pH 5.5 and ionic strength of 10^−2^ M is shown in Fig. 1. These experimental results were interpreted in terms of the 3D electrokinetic model developed in Adamczyk et al. (2010). The following analytical expression was derived for calculating the zeta potential of polyelectrolyte covered surfaces:

7 $$\zeta (\Uptheta ) = F_{i} (\Uptheta )\zeta_{i} + F_{\text{p}} (\Uptheta )\zeta_{\text{p}} ,$$

where *ζ*(*Θ*) is the zeta potential of the polyelectrolyte-covered substrate; *ζ*_*i*_ is the zeta potential of bare mica; *ζ*_p_ is the zeta potential of PAH molecules in the bulk, and $F_{i} \left( \Uptheta \right),\,F_{\text{p}} \left( \Uptheta \right)$ are the dimensionless functions of the coverage and the electrical double-layer thickness. In the case of thin double layers, the *C*_*i*_, *C*_p_ approach the limiting values of $C_{i}^{0} = 10.2$ and $C_{p}^{0} = 6.51$, respectively (Adamczyk et al. 2010). As shown in Wasilewska and Adamczyk (2011), the $F_{i} \left( \Uptheta \right),\,F_{\text{p}} \left( \Uptheta \right)$ functions can be approximated by the following analytical expressions:

8 $$F_{i} (\Uptheta ) = e^{{ - C_{i} \Uptheta }}$$

$$F_{\text{p}} (\Uptheta ) = \frac{1}{\sqrt 2 }(1 - e^{{ - \sqrt 2 C_{\text{p}} {\kern 1pt} \Uptheta }} ).$$

Obviously, for bare surfaces, where *θ* = 0, *F*_*i*_(*θ*) = 1, and *F*_p_(*θ*) = 1. On the other hand, for high coverage range, the *F*_*i*_(*θ*) function vanishes, and *F*_p_(*θ*) tends to $\frac{1}{\sqrt 2 }$. Thus, using Eq. (8), one can deduce that the limiting zeta potential for surfaces covered by particles is given by

9 $$\zeta_{\infty } = \zeta_{\text{p}} /\sqrt 2 = 0.71\zeta_{\text{p}} .$$

Theoretic results calculated using Eqs. (7, 8) are plotted in Fig. 1 as a solid line. As can be seen, they reflect well the experimental data for the entire range of PAH coverage. Analogous results obtained for other ionic strength and pH are discussed in Morga and Adamczyk (2013).

**Fig. 2:**
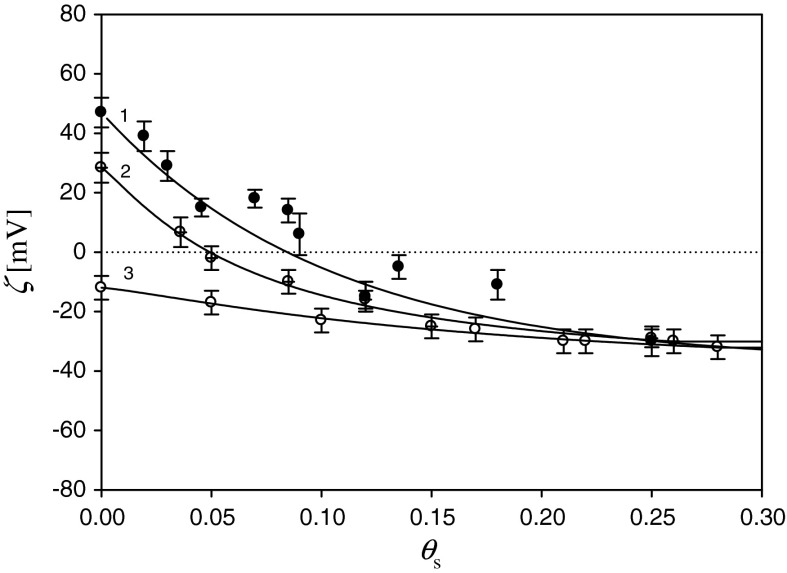
The dependencies of zeta potential of silver monolayers on the coverage *θ*_s_ determined via the streaming potential measurements for various PAH supporting coverages. Silver nanoparticle adsorption was carried out for pH 6, *I* = 10^−2^ M, *T* = 298 K, *1* (*black circle*) *θ*_PAH_ = 0.5, *2* (*circled plus*) *θ*_PAH_ = 0.17, *3* (*white* *circle*) *θ*_PAH_ = 0.07

After fully characterizing PAH monolayers on mica, a series of experiments was performed to determine the formation of silver nanoparticle monolayers. Hence, in the first stage, the supporting PAH monolayer of a desired coverage was formed. Afterward, the silver monolayer was formed under diffusion-controlled transport using suspensions of appropriate concentration. The coverage of silver particles was calculated from Eq. (6) using the characteristic cross-section area of 615 nm^2^ (see Table 1). In the first series of experiments, the influence of the supporting PAH layer density on the silver monolayer formation was studied. Typical results of these experiments, performed for *I* = 10^−2 ^M, pH 5.5, and various supporting layer coverages, *θ*_PAH_ ranging from 0.07 to 0.5 are shown in Fig. 2.

As can be seen, in all cases, the decrease in the zeta potential of the supporting PAH layer upon silver particle deposition was observed, even for *θ*_PAH_ = 0.07. This is a rather unusual behavior, contradicting the mean-field DLVO theory, because negatively charged particles are deposited on negatively charged (on average) substrate. However, such deviations were often observed for colloid particle adsorption on protein supporting layers, e.g., fibrinogen (Adamczyk et al. 2011a) and albumin (Jachimska et al. 2012). They were quantitatively explained in terms of the heterogeneous charge distribution in the supporting layer stemming from molecule density fluctuations. This leads to formation of local adsorption sites exhibiting positive charge in contrast to the average negative charge of substrates.

As can be seen in Fig. 2, for higher PAH coverage, the variations in the zeta potential of silver monolayers are significantly more pronounced. This results in an abrupt decrease in the zeta potential that can be exploited for a sensitive monitoring of particle deposition process. It is interesting to mention that in all cases, the limiting zeta potential of silver particle monolayers approaches −29 mV. Considering that the bulk value of the silver particle zeta potential is −39 mV, one can deduce that this agrees with the theoretic value predicted from Eq. (9). It is also interesting to mention that the influence of the mica zeta potential and the supporting PAH layer on the final zeta potential of the silver monolayer is rather negligible. An analogous behavior was previously observed for the colloid particle bilayers at mica formed by deposition of positive amidine latex particles and negative polystyrene latex particles (Zaucha et al. 2011).

**Fig. 3:**
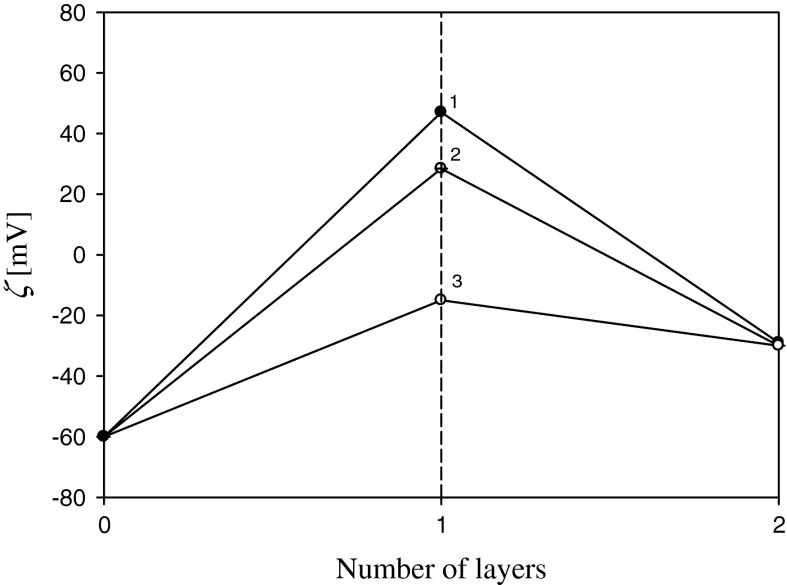
Formation of the silver nanoparticle monolayer on PAH-covered mica shown as the dependence of the zeta potential on the layer number (the deposition conditions: pH 6, *I* = 10^−2^ M, *T* = 298 K). *1* (*black circle*) *θ*_PAH_ = 0.5, *θ*_s_ = 0.28; *2* (*circled plus*) *θ*_PAH_ = 0.17, *θ*_s_ = 0.28; *3* (*white circle*) *θ*_PAH_ = 0.07, *θ*_s_ = 0.28

Since these results have a major significance for determining the effect of substrate and the supporting layer of polyelectrolyte films formation according in the widely used layer by layer (LbL) technique (Zaucha et al. 2011), we have presented them in Fig. 3, in the form of the “saw-like” graph.

As can be seen, even for the supporting layer coverage as low as 0.07, the electrokinetic properties of silver monolayer become fixed; that is, the zeta potential of the silver monolayer assumes 0.7 of the bulk zeta potential.

**Fig. 4:**
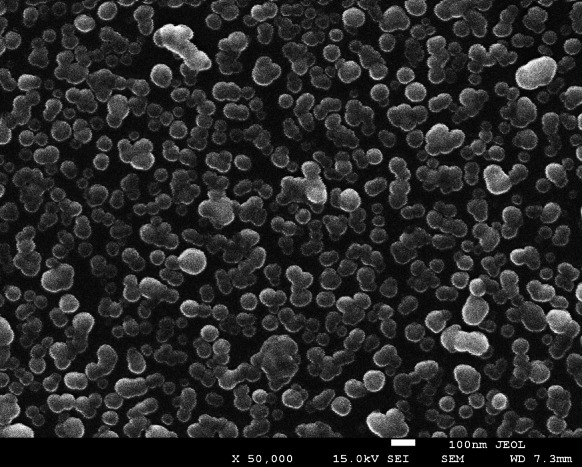
A monolayer of silver particles derived from SEM imaging *θ*_s_ = 0.28

In order to estimate, the validity of Eq. (6), which was used to calculate the nominal coverage of silver particles, a series of experiments was performed where the true coverage was determined by SEM imaging. According to this procedure, after completing the streaming potential measurements, the mica sheets covered by silver particles were dried and transferred to the SEM chamber. A monolayer obtained for various PAH-supporting layers is shown in Fig. 4. The coverage of particles was calculated by a direct counting of their number over equal-sized surface areas chosen at random over the mica sheet. The total number of particle counted in this way was ca. 1,000 that ensures a relative accuracy of this measurement better that 3 %.

In the next two series of experiments, the influence of the ionic strength and pH on the silver particle monolayer formation was systematically studied. In all these experiments, the dense PAH monolayer, having the coverage of 0.5, was used.

**Fig. 5:**
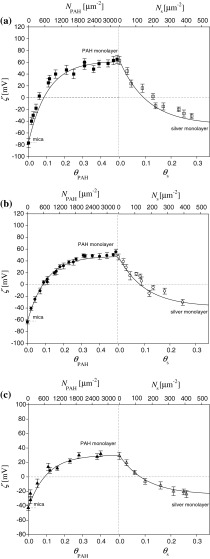
The dependence of the zeta potential of mica on the coverage of PAH and silver nanoparticles. The *points* denote experimental results obtained from the streaming potential measurements. The deposition conditions *I* = 10^−2 ^M NaCl, pH 6, and *T* = 298 K. Streaming potential measurements carried out at pH 5.5 and various ionic strengths: **a** *I* = 10^−3 ^M NaCl, **b** *I* = 10^−2 ^M NaCl, **c** *I* = 0.15 M NaCl. The *solid lines* denote the exact theoretic results calculated from the 3D electrokinetic model, Eqs. (7, 8)

**Fig. 6:**
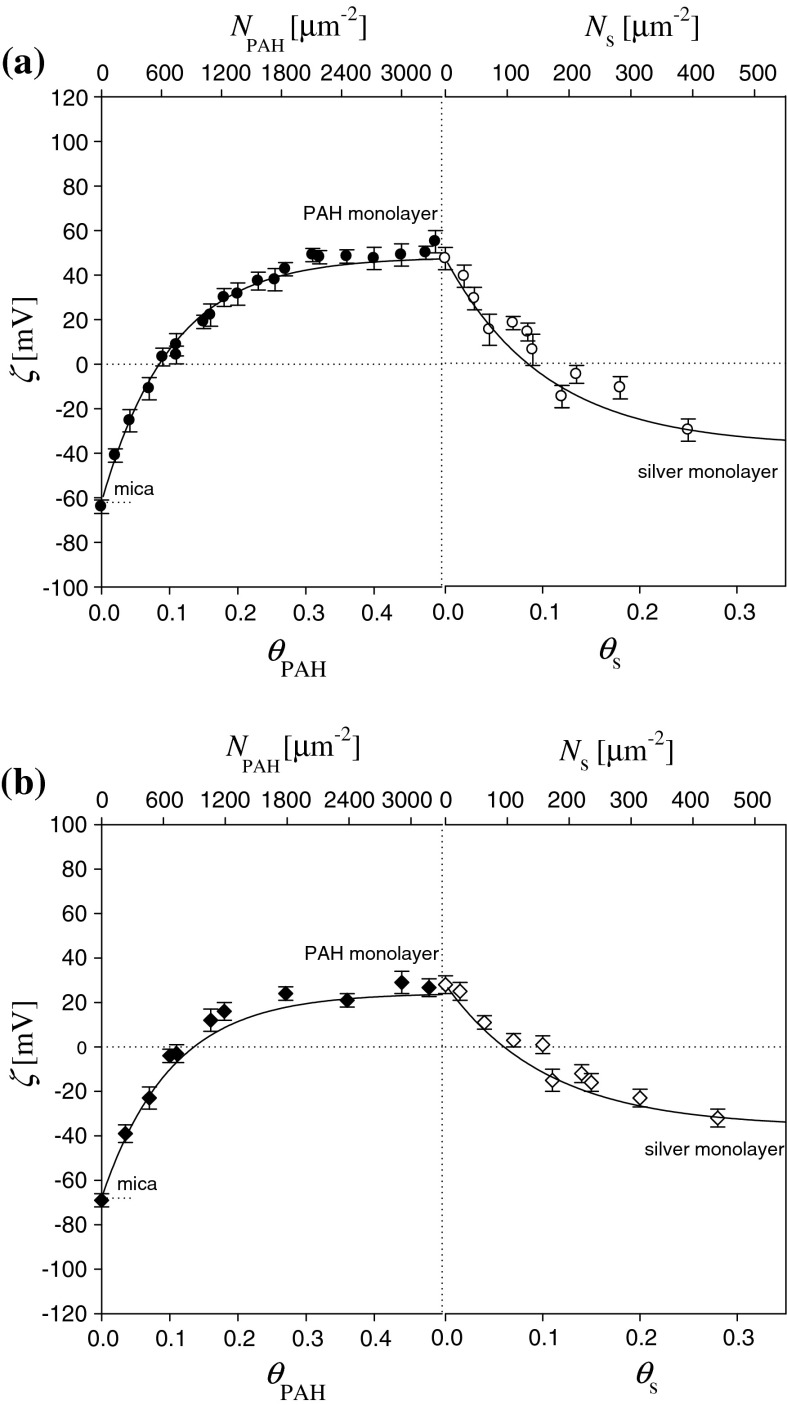
The dependence of the zeta potential of mica on the coverage of PAH and silver nanoparticles. The *points* denote experimental results obtained from the streaming potential measurements. The deposition conditions *I* = 10^−2 ^M NaCl, pH 6, and *T* = 298 K. Streaming potential measurements carried out at fixed ionic strength *I* = 10^−2 ^M NaCl and different pH: **a** pH 5.5, **b** pH 9.0. The *solid lines* denote the exact theoretic results calculated from the 3D electrokinetic model, Eqs. (7, 8)

Results of such measurements obtained for various ionic strength are shown in Figs. 5 and 6. The experimental results were interpreted in terms of electrokinetic model where both the molecules and particles adsorbed at the solid/liquid interface were treated as isolated entities exhibiting a 3D charge distribution (Adamczyk et al. 2010, 2011b; Morga et al. 2012).

As can be seen in Fig. 5, the formation of silver particle monolayers results in an abrupt decrease in the surface zeta potential with the slope *ζ* versus *θ* considerably exceeding 10 for *θ* below 0.1. The inversion in sign of the zeta potential is observed for *θ*_s_ = 0.08. For higher coverage of silver particles (*θ*_s_ > 0.1), the zeta potential variations become rather minor, and for *θ*_s_ > 0.25, the zeta potential of silver monolayers attains asymptotic values of −32, −27, and −23 mV for ionic strengths 10^−3^, 10^−2^ and 0.15 M NaCl, respectively. Thus, it was confirmed in these measurements that the limiting zeta potential for the high coverage range of silver nanoparticles approaches 1/√2 = 0.71 of the bulk zeta potential of the nanoparticles.

In the second series of studies, the dependence of the zeta potential on the coverage of silver nanoparticles under different pH was studied. Typical results of these experiments, performed for *I* = 10^−2 ^M pH 5.5 and 9.0, *T* = 298 K are shown in Fig. 6.

As can be see seen, the formation of a silver monolayer results in an abrupt decreases in the zeta potential with the slope *ζ*_s_ versus *θ*_s_ considerably exceeding 10 for *θ* close to 0.1. For higher coverage of silver particles (*θ* > 0.1), the zeta potential variations become rather minor, and for *θ*_*s*_ > 0.25, the zeta potentials of the silver layer attain the asymptotic value of −27 and −35 mV for pH 5.5 and 9.0, respectively that are close (within experimental error bounds) to the theoretic values of 1/√2 = 0.71 of the bulk zeta potential of the nanoparticles (see Table 2).

### Kinetics of silver nanoparticle release—streaming potential studies

**Fig. 7:**
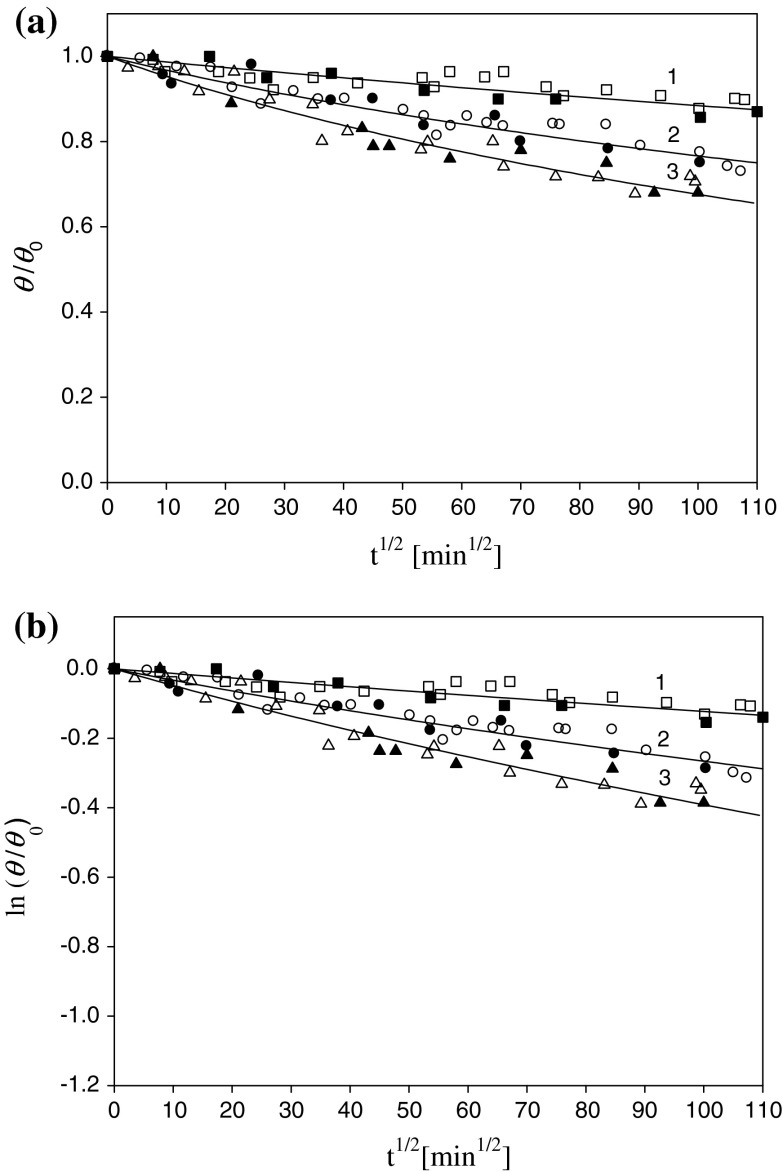
The kinetics of silver particle release expressed as **a** the dependence of *θ/θ*_0_ on *t* ^1/2^, **b** the dependence of ln(*θ/θ*_0_) on *t* ^1/2^. Ionic strengths: *1* (*black square*) 10^−4 ^M, *2* (*black circle*) 10^−2 ^M, *3* (*black triangle*) 0.1 M NaCl. The *full points* denote experimental data obtained by the streaming potential method (the initial coverage of particles *θ*_0_ = 0.20). The *hollow points* denote SEM, *ex situ* measurements. The deposition conditions *I* = 10^−2 ^M NaCl, pH 6, *T* = 298 K. The *solid lines* denote the theoretic results calculated from the RSA model by the numerical integration of Eq. (10)

In order to determine the stability of silver monolayers obtained as described above, a few series of desorption experiments were performed. Firstly, silver particle monolayers of a defined initial coverage were produced at pH 5.5 and *I* = 10^−2 ^M, in the electrokinetic cell. As mentioned above, the initial coverage was adjusted by changing the suspension concentration and adsorption time. Afterward, the cell was flushed with pure electrolyte of appropriate ionic strength (10^−4^–0.1 M NaCl) and pH, and the particles were allowed to desorb under diffusion-controlled transport for a desired period of time up to 200 h. The final coverage of the silver nanoparticles remaining on the surface was determined in situ by direct streaming potential measurements. After the in situ measurements, the coverage of particles was also determined from SEM micrographs by a direct enumeration procedure. Although this method is tedious and time-consuming, it furnishes reliable data of high precision. Typical release kinetics runs obtained for the initial coverage of silver particles equal to *θ*_0_ = 0.20 for various ionic strengths are presented in Fig. 7.

As can be seen in Fig. 7, the particle release rate increases with ionic strength. Thus, for *I* = 10^−4^ M, the residue coverage of the monolayer after the time of 200 h is 90 % of the initial value, whereas for *I* = 0.1 M, the coverage decreases to 65 % of the initial value. Thus, for low ionic strength, the particle release can be considered as practically negligible.

**Fig. 8:**
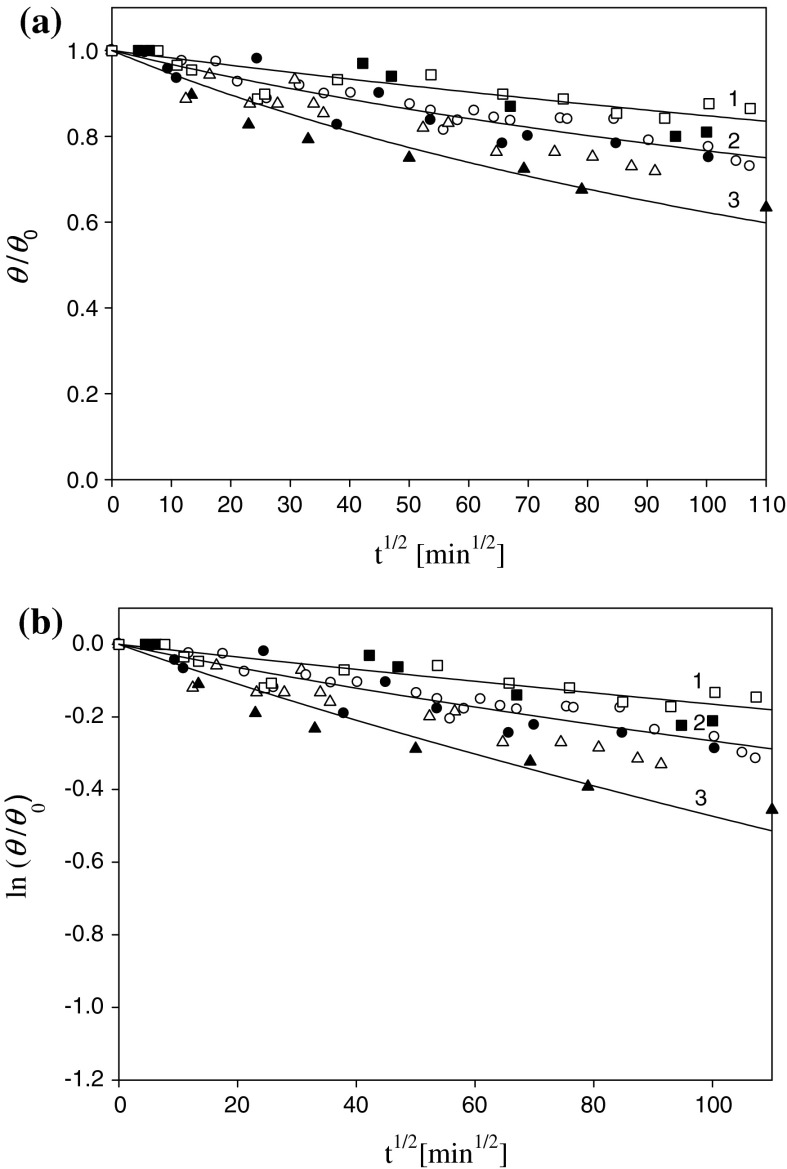
The kinetics of silver particle desorption expressed as **a** the dependence of *θ/θ*_0_ on *t* ^1/2^, **b** the dependence of ln (*θ/θ*_0_) on *t* ^1/2^ and pH *1* (*black square*) 9.0, *2* (*black circle*) 5.5, *3* (*black triangle*) 3.5 (*I* = 10^−2 ^M, *T* = 298 K). The *full points* denote experimental data obtained by the streaming potential in situ method (the initial coverage of particles *θ*_0_ = 0.20). The *hollow points* denote SEM, *ex situ* measurements. The deposition conditions *I* = 10^−2 ^M NaCl, pH 6, *T* = 298 K. The *solid lines* denote the theoretic results calculated from the RSA model by the numerical integration of Eq. (10)

Additionally, a series of experiments was performed in order to investigate the dependence of silver nanoparticle release rate on pH. Typical kinetic runs obtained for *I* = 10^−2 ^M, *θ* = 0.20, and pH 3.5, 5.5, and 9.0 are shown in Fig. 8.

As can be seen, the nanoparticle release rate decreases with pH. Thus, for pH 3.5, the residue coverage of the monolayer after the time of 200 h is 62 % of the initial value, whereas for pH 5.5 and 9.0, it remains at the level of 75 and 83 %, respectively.

The experimental runs shown in Figs. 7 and 8 are quantitatively interpreted in terms of the RSA model previously developed (Adamczyk 2006; Oćwieja et al. 2012a). The kinetic equation describing the changes in the particle coverage with the adsorption time is as follows:

10 $$\int\limits_{{\theta_{0} }}^{\theta } {\frac{{B(\theta^{'} ){\text{d}}\theta^{'} }}{{\theta^{'} }}} { = } - \frac{2}{{K_{a} }}\left( {\frac{Dt}{\pi }} \right)^{1/2} \, ,$$

where *B*(*θ*) is the surface blocking function, and *K*_a_ is the equilibrium adsorption constant.

For spherical particles, the RSA blocking function can be approximated by the expression:

11 $$B(\theta ) = (1 + a_{1} \overline{\theta } + a_{2} \overline{\theta }^{2} + a_{3} \overline{\theta }^{3} )(1 - \overline{\theta } )^{3} ,$$

where $\overline{\theta } = \theta /\theta_{\text{mx}}$ is the normalized coverage of particles; *θ*_mx_ is the maximum coverage of particles equal to 0.547 for hard (non-interacting) particles (Adamczyk 2006), and *a*_1_–*a*_3_ are the dimensionless coefficients equal to 0.812, 0.426, and 0.0717, respectively.

In the general case, for arbitrary initial coverage, Eq. (10) can be evaluated by numerical integration. Using such solutions to fit experimental data, one can calculate the equilibrium adsorption constants *K*_a_ for various particle sizes. The adsorption constants are collected in Tables 3 and 4.

**Table 3:** The equilibrium adsorption constants *K*_a_ and the energy minima *ϕ*_m_ for pH 5.5 and various ionic strengths

	*I* = 10^−4^ M		*I* = 10^−2^ M		*I* = 0.1 M	
	*K*_a_ (cm)	*ϕ*_m_ (kT)	*K*_a_ (cm)	*ϕ*_m_ (kT)	*K*_a_ (cm)	*ϕ*_m_ (kT)
In situ measurements	8 ± 2	−20	4.1 ± 0.8	−19	2.0 ± 0.4	−18
*Ex situ* measurements	5.0 ± 1	−19	1.9 ± 0.4	−18	1.1 ± 0.2	−18

**Table 4:** The equilibrium adsorption constants *K*_a_ and the energy minima *ϕ*_m_ for *I* = 10^−2^ M and various pH

	pH 3.5		pH 5.5		pH 9.0	
	*K*_a_ (cm)	*ϕ*_m_ (kT)	*K*_a_ (cm)	*ϕ*_m_ (kT)	*K*_a_ (cm)	*ϕ*_m_ (kT)
In situ measurements	1.3 ± 0.3	−18	4.1 ± 0.8	−19	4.2 ± 1	−19
*Ex situ* measurements	1.3 ± 0.3	−18	1.9 ± 0.2	−18	3.0 ± 0.6	−19

Knowing the equilibrium adsorption constants one can determine the energy minima depth using the equation previously derived (Oćwieja et al. 2012a):

12 $$K_{\text{a}} = \delta_{\text{m}} \left( {\frac{\pi kT}{{\left| {\phi_{\text{m}} } \right|}}} \right)^{1/2} e^{{ - \phi_{\text{m}} /kT}} ,$$

where *δ*_m_ is the characteristic thickness of the energy minima between the particle and the interface.

Equation (12) can be iteratively solved, which results in the approximate expression:

13 $$\phi_{\text{m}} /kT = - \ln \frac{{K_{\text{a}} }}{{\delta_{\text{m}} }} - \frac{1}{2}\ln \left( {\frac{{\ln \frac{{K_{\text{a}} }}{{\delta_{\text{m}} }}}}{\pi }} \right).$$

Assuming a typical value of the energy minimum *δ*_m_ = 0.5 nm = 5 × 10^−7^ cm, one obtains, from Eq. (13), the following energy minima depths: *ϕ*_m_ = −20 kT for 10^−4^ M, *ϕ*_m_ = −19 kT for 10^−2^ M, and *ϕ*_m_ = −18 kT for 0.1 M. Similar results were obtained for *ex situ* measurements, see Table 3. It is worth mentioning that the energy minima depth increases slightly with ionic strength. However, the difference between ionic strength of 10^−4^ and 10^−2^ M is only −1 kT for in situ and *ex situ* measurements.

Analogous results were obtained in experiments performed for various pH and fixed ionic strength of 10^−2^ M where *ϕ*_m_ varies between −18 kT for pH 3.5 and −19 kT for pH 9.0. In this case, the agreement between the streaming potential and the *ex situ* method was even better than before (see Table 4).

**Fig. 9:**
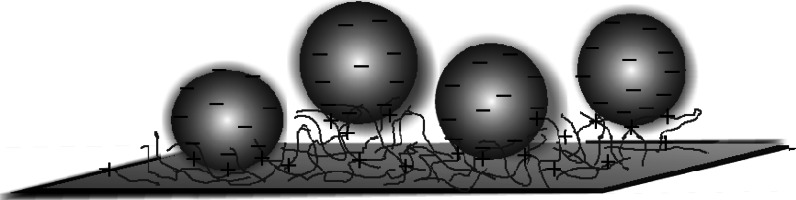
A schematic view of silver nanoparticles immobilization on PAH-covered mica

It should be mentioned that the minor changes in the energy minima depths with the ionic strength and pH determined in our work agree with the discrete charge interaction model previously proposed in Adamczyk (2006). According to this concept, the electrostatic interactions are governed by the finite number of ion pairs, *N*_i_, formed between the PAH molecules and silver particles (see Fig. 9), strictly related to the number of charges on the silver particle. It is assumed (Adamczyk 2006) that these interactions can be described by the unscreened Coulomb potential, i.e.,

14 $$\phi_{m} = - N_{i} \frac{{e^{2} }}{{4\pi \varepsilon d_{im} }},$$

where *N*_*i*_ is the number of ion pairs in the interaction zone, and *d*_im_ is the minimum distance charges in the between ion pairs.

It is interesting to observe that the interaction energy expressed by Eq. (14) is independent of the particle size and the ionic strength.

Using this ion pair interaction model, one can reasonably reflect the experimentally determined interaction energy depths. Thus, for *I* = 10^−4^ M, *T* = 298 K, assuming *N*_*i*_ = 16 (the number of the available charges on the silver particles in the interaction zone) and *d*_im_ = 0.5 nm, one can calculate from Eq. (16) *ϕ*_m_ = −22 kT, which is close to the experimental value of −20 kT. Analogously, for *I* = 10^−2^ M, assuming *N*_*i*_ = 10, one obtains from Eq. (16) *ϕ*_m_ = −16 kT, which is also close to the experimental value of −19 kT. Analogous results were observed for *I* = 10^−2^ M and different pH. In the case of pH 3.5, the value of energy minima depth calculated from the ion pair model was *ϕ*_m_ = −17 kT (experimental value −18 kT), and for pH 9.0, *ϕ*_m_ = −18 kT (experimental value −19 kT).

As can be deduced, the ion pair model well reflects the basic features observed in the silver particle release kinetics measurements, i.e., the negligible dependence of the depth of energy minima on ionic strength and pH.

## Conclusions

It was demonstrated that the zeta potential of the silver monolayers on PAH-covered mica can be effectively determined via streaming potential measurements. The experiments conducted in this way are adequately interpreted via the electrokinetic model expressed by Eqs. (7, 8). This is significant for basic science because the validity of the electrokinetic model to interpret the behavior of real nanoparticle systems is confirmed.

Additionally, it was shown that the in situ streaming potential measurements can be exploited for quantitatively studying particle release from monolayers. This enabled one to determine the equilibrium adsorption constants *K*_a_, which is a prohibitive task using other experimental methods. Knowing *K*_a_, the binding energies of silver particles *ϕ*_m_ (energy minima depths) can be calculated using Eq. (13) for various physicochemical conditions. It was shown that the energy minima varied between −20 and −18 kT being practically independent of ionic strength and pH. These experimental evidences, representing first of this kind in the literature, indicate that the silver particle release kinetics was governed by the electrostatic interactions among ion pairs.

It was also shown that it is feasible to quantitatively monitor the formation of silver particle monolayers via the streaming potential measurements, which is considerably more convenient than by using the tedious and time-consuming *ex situ* measurements.

Such monolayers may find practical applications as universal substrates for protein immobilization (biosensors) and in catalytic applications.
